# CNRS School "Nanophysics for Health", 5-9 November 2012, Mittelwhir, France

**DOI:** 10.1186/1477-3155-11-S1-I1

**Published:** 2013-12-10

**Authors:** Jérémie Léonard, Didier Rouxel, Pascal Hébraud

**Affiliations:** 1 Institut de Physique et Chimie des Matériaux de Strasbourg & Labex NIE, Université de Strasbourg, CNRS UMR 7504, F-67034 Strasbourg Cedex 2, France; 2 Institut Jean Lamour, CNRS UMR 7198, Université de Lorraine, F- 54506 Vandoeuvre-lès-Nancy Cedex, France

## Acknowledgements

This supplement to *Journal of Nanobiotechnology *gathers eight tutorials corresponding to the topics of eight lectures given at the CNRS School entitled "Nanophysics for Health", which took place in Mittelwhir, France, on 5 - 9 November 2012. The scope of the school was the implementation and relevance of tools and concepts from nanophysics applied to biological sciences and biomedical technologies. The onset of new, powerful methods for the investigation of biomolecular or cellular systems, as well as the development of innovative technologies for diagnosis or therapy are among the great promises of nanotechnologies in general, and of nanophysics in particular. This field of research is very dynamic and intrinsically multidisciplinary. It requires a tight collaboration between partners from different scientific horizons, which is favored by their sharing common, multidisciplinary scientific and technological skills. The goal of the school was to contribute to the building of this common scientific background and language.

Fifty scientists (including lecturers) with diverse backgrounds in nanoscience, physics, biophysics, biology or chemistry attended the school. The program was essentially composed of keynote lectures focusing on three main topics: Nanophysics for Molecular Biology, Nanophysics for Cellular Biology, and Nanophysics for Diagnosis and Therapy. Practical sessions focusing on the use of Atomic Force Microscopy for the investigation of biological samples were also organized. In total, twelve 2-hour lectures were delivered, eight of them being published as tutorials in the present supplement of *Journal of Nanobiotechnology*.

An introductory lecture (by D. Riveline) did present the manipulation of single molecules, and in particular the reasons why single molecule observation and manipulation are a unique tool to understand the dynamical properties of macromolecules. Then, a set of lectures was devoted to the structure, dynamics and dynamical assembly of proteins, including experimental developments in the firled of single molecule spectroscopy (B. Schuler), atomic force microscopy (F. Rico), and 3-dimensional imaging with electron microscopy (P. Schultz) as well as theoretical developments (K. Kruse). The last set of lectures showed that manufactured nanoparticles are very promising tools for nanomedicine, in the field of therapy (by P. Couvreur), as well as imaging and diagnosis (by F. Gazeau, L. Bonacina).

For the school organizing committee, the guest editors: Jérémie Léonard, Didier Rouxel, Pascal Hébraud.

## Competing interests

The authors declare that they have no competing interests.

## Notes

This article has been published as part of *Journal of Nanobiotechnology *Volume 11 Supplement 1, 2013: Nanophysics for Health. The full contents of the supplement are available online at http://www.jnanobiotechnology.com/supplements/11/S1. Publication charges for this tutorial were funded by the CNRS School "Nanophysics for Health", 5 - 9 November 2012, Mittelwhir, France

